# Genome-Wide Association Study Identified a Narrow Chromosome 1 Region Associated with Chicken Growth Traits

**DOI:** 10.1371/journal.pone.0030910

**Published:** 2012-02-16

**Authors:** Liang Xie, Chenglong Luo, Chengguang Zhang, Rong Zhang, Jun Tang, Qinghua Nie, Li Ma, Xiaoxiang Hu, Ning Li, Yang Da, Xiquan Zhang

**Affiliations:** 1 Guangdong Provincial Key Lab of Agro-Animal Genomics and Molecular Breeding, Guangzhou, Guangdong, China; 2 Department of Animal Science, University of Minnesota, Saint Paul, Minnesota, United States of America; 3 State Key Laboratory of Livestock and Poultry Breeding, Guangzhou, Guangdong, China; 4 Institute of Animal Science & Veterinary, Hainan Academy of Agricultural Sciences, Haikou, Hainan, China; 5 College of Biological Science, China Agricultural University, Beijing, China; Auburn University, United States of America

## Abstract

Chicken growth traits are important economic traits in broilers. A large number of studies are available on finding genetic factors affecting chicken growth. However, most of these studies identified chromosome regions containing putative quantitative trait loci and finding causal mutations is still a challenge. In this genome-wide association study (GWAS), we identified a narrow 1.5 Mb region (173.5–175 Mb) of chicken (*Gallus gallus*) chromosome (GGA) 1 to be strongly associated with chicken growth using 47,678 SNPs and 489 F2 chickens. The growth traits included aggregate body weight (BW) at 0–90 d of age measured weekly, biweekly average daily gains (ADG) derived from weekly body weight, and breast muscle weight (BMW), leg muscle weight (LMW) and wing weight (WW) at 90 d of age. Five SNPs in the 1.5 Mb *KPNA3*-*FOXO1A* region at GGA1 had the highest significant effects for all growth traits in this study, including a SNP at 8.9 Kb upstream of *FOXO1A* for BW at 22–48 d and 70 d, a SNP at 1.9 Kb downstream of *FOXO1A* for WW, a SNP at 20.9 Kb downstream of ENSGALG00000022732 for ADG at 29–42 d, a SNP in *INTS6* for BW at 90 d, and a SNP in *KPNA3* for BMW and LMW. The 1.5 Mb *KPNA3*-*FOXO1A* region contained two microRNA genes that could bind to messenger ribonucleic acid (mRNA) of *IGF1*, *FOXO1A* and *KPNA3*. It was further indicated that the 1.5 Mb GGA1 region had the strongest effects on chicken growth during 22–42 d.

## Introduction

Growth traits play important roles in studying animal developments. During the last two decades, many quantitative trait loci (QTL) underlying growth were identified [1]–[17]. The chicken QTL database [3] have over 1500 QTL associated with growth traits with QTL locations on the entire genome except chromosome 21, 22, 25 and W. Most QTL are located on macrochromosomes including chicken (*Gallus gallus*) chromosome (GGA) 1, 2, 3, 4, and Z. Although great advances have been achieved, most of the reported QTLs were mapped with low-density microsatellite markers that were inadequate for further fine mapping analysis [18]–[20]. Genome-wide association study (GWAS) that uses single nucleotide polymorphism (SNP) panel covering the entire chicken genome improves to great extent the mapping accuracy due to the dense genome coverage unavailable from microsatellite markers. In this study, we conducted GWAS using 47,678 SNPs for chicken body growth traits from hatching to 90 d of age in an F2 population derived from reciprocal cross between White Recessive Rock (WRR) and Xinghua (XH) chickens.

## Results

### SNP effects on growth traits

**Figure 1:**
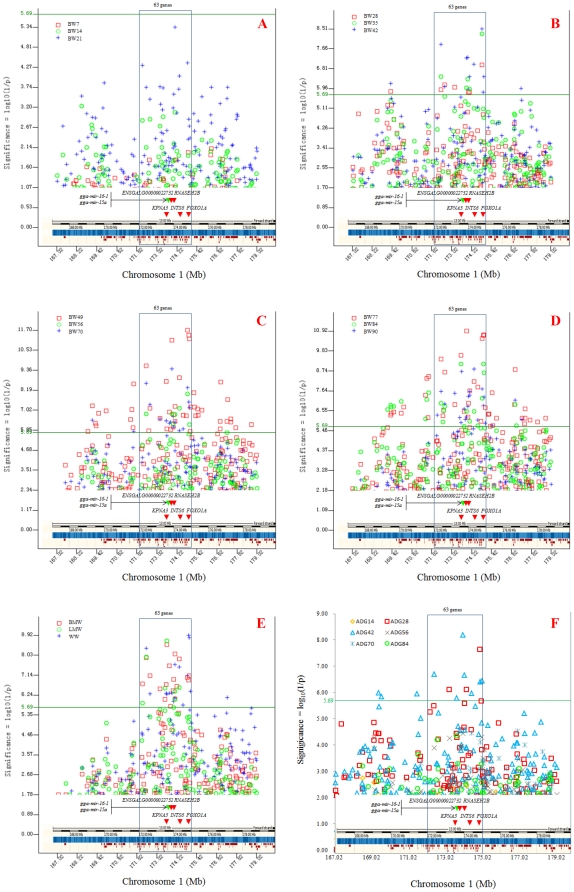
SNP effects on chicken growth traits in the 167–179 Mb region of GGA1. The green line was the 5% Bonferroni genome-wide significance threshold. The rectangular box with blue border framed the 3 Mb region of GGA1 (172–175 Mb) with the most significant effects for all 23 traits. Red arrows highlight genes and the green arrows are the two non-coding RNA genes.

A total of 257 SNP effects involving 68 SNPs and 23 genes were detected for 18 of the 23 traits with genome-wide significance (*P<*2.04×10^−6^) (Table 1, Figure S1). All except nine of the 257 SNP effects were located in the 167–179 Mb region of GGA1 (Table S1, Figure 1A–1D). Other than this GGA1 region, only nine SNP effects reached genome-wide significance, including four effects of a GGA4 SNP for BW90, BW70, BW56, and ADG56, two effects of a GGA2 SNP for BMW and WW, one effect on GGA18 for BW49, one effect of a GGA19 SNP for BW63, and one effect of a GGA1 SNP for WW (Table 1). However, these effects were far less significant than those in the 167–179 Mb region of GGA1 (Figure S1). No SNP effect reached genome-wide significance for early growth traits, including BW0, BW7, BW14, BW21, and ADG14.

**Table 1:** Distribution of results reached genome-wide significance (*P<*2.04×10^−6^) for each of the 23 growth traits by chromosome.

Trait^a^	Chr1	Chr2	Chr4	Chr18	Chr19	Total
BW0	0	0	0	0	0	0
BW7	0	0	0	0	0	0
BW14	0	0	0	0	0	0
BW21	0	0	0	0	0	0
BW28	9	0	0	0	0	9
BW35	5	0	0	0	0	5
BW42	12	0	0	0	0	12
BW49	7	0	0	1	0	8
BW56	18	0	1	0	0	19
BW63^b^	1	0	0	0	1	2
BW70	49	0	1	0	0	50
BW77	43	0	0	0	0	43
BW84	26	0	0	0	0	26
BW90	23	0	1	0	0	24
ADG14	0	0	0	0	0	0
ADG28	3	0	0	0	0	3
ADG42	10	0	0	0	0	10
ADG56	0	0	1	0	0	1
ADG70	0	0	0	0	0	0
ADG84	0	0	0	0	0	0
BMW	18	1	0	0	0	19
LMW	7	0	0	0	0	7
WW	18	1	0	0	0	19
SUM	249	2	4	1	1	257

a BW0, BW7, BW14, BW21, BW28, BW35, BW42, BW49, BW56, BW63, BW70, BW77, BW84, and BW90 represented body weight at hatching, 7, 14, 21, 28, 35, 42, 49, 56, 63, 70, 77, 84 and 90 d of age, respectively. ADG14, ADG28, ADG42, ADG56, ADG70, and ADG84 represented average daily gain at 1–14 d, 15–28 d, 29–42 d, 43–56 d, 57–70 d, and 71–84 d, respectively. BMW, LMW, and WW represented breast muscle weight, leg muscle weight and wing weight, respectively.

b For BW63, only 224 of the 489 individuals had trait observations.

Analysis of biweekly average daily gains (ADG) aimed at identifying SNP effects associated with net body growth for a given time period. The results showed that the 3 Mb region of 172–175 Mb had the largest numbers of significant SNP effects (10 out of 13) and had the most significant effects for ADG28 and ADG42. The most significant effect was from a SNP at 8.9 Kb upstream of the *forkhead box O1A* gene (*FOXO1A*) for ADG28 and a SNP at 20.89 Kb downstream of ENSGALG00000022732 for ADG42 (Table 2). For early growth prior to 21 d of age (BW0, BW7, BW14, BW21, and ADG14), no significant SNP effect was detected on any chromosome. For net growth beyond 42 d of age (ADG56, ADG70, and ADG84), no GGA1 effect was detected but one SNP effect on GGA4 was significant for ADG56 (Table 1). The results of ADG analysis indicated that the GGA1 effects on chicken body growth were the strongest in the time period of 22–42 d after hatching, which is an important time period for commercial broilers. The most significant effects for BMW and LMW were from a SNP in the *karyopherin alpha* 3 gene (*KPNA3*) and that for WW was from a SNP at 8.9 Kb upstream of *FOXO1A* for several BW and ADG traits (Table 2).

**Table 2:** Five SNP markers on GGA1 with most significant effects for chicken growth traits.

SNP	Allele	Position (bp)	Nearest Gene	ADG28	ADG42	BW90	BMW	LMW	WW
rs15497910	A/G	173613981	*KPNA3*	5.30E-05	5.68E-03	2.05E-09	**3.22E-09**	**2.09E-09**	5.20E-07
rs13972304	A/C	173931557	20.9 Kb D *ENSGALG00000022732*	3.73E-04	**6.39E-09**	6.27E-07	2.06E-05	1.29E-04	4.57E-07
rs14917647	A/C	174379124	*INTS6*	2.65E-05	1.06E-06	**1.44E-09**	1.36E-08	2.62E-07	3.09E-08
rs13973515	C/T	174847719	8.9 Kb U *FOXO1A*	**2.34E-08**	4.00E-07	1.83E-08	9.47E-08	5.69E-06	7.82E-08
GGaluGA055359	A/G	174921993	1.9 Kb D *FOXO1A*	2.17E-06	1.46E-05	1.49E-07	1.24E-07	8.87E-06	**1.20E-09**

Bold face indicates the most significant SNP effect for the trait. U = upstream. D = downstream. ADG28 and ADG42 represented average daily gain at 15–28 d and 29–42 d, respectively. BW90 represented body weight at 90 d of age. BMW, LMW, and WW represented breast muscle weight, leg muscle weight and wing weight, respectively.

The association results revealed that all the most significant effects for all growth traits in this study involved only five SNPs in a narrow 1.5 Mb region of 173.5∼175 Mb on GGA1, including the four SNPs discussed above. The fifth SNP was in the *integrator complex subunit 6* gene (*INTS6*) (Table 2) and was the most significant SNP for aggregate BW90.

### Evidence from allele frequencies and linkage disequilibrium (LD)

As secondary evidence of SNP effects on growth traits, frequencies of favorable and unfavorable alleles of the 15 significant SNPs in the 1.5 Mb region were compared in four chicken populations with divergent body growth rates: two populations of White Recessive Rock (WRR and WRR1), and two Chinese breeds, XH and Bai Er Huang (BEH). The WRR and WRR1 chickens were fast-growing broilers while XH and BEH are slow-growing chickens. We hypothesized that SNPs relevant to growth had higher frequencies in the fast-growing group than in the slow-growing group.

Of the 15 SNPs, twelve (including the five SNPs with most significant effects for all growth traits in this study) had higher frequencies of the favorable alleles in the fast-growing group than in the slow-growing group and the between-group frequency differences were significant (*P*<0.0033, Table S2), except that one SNP at 173,593,810 bp was insignificant between WRR and XH (*P* = 0.0127, Table 3). Three SNPs in the slow-growing group and five SNPs in the fast-growing group (marked in green color in Table S2) had significant within-group frequency differences, but they were far less significant than the between-group differences. These results of frequency differences indicated that the 14 SNPs could either be a part of a causal mechanism or in coupling linkage phase with causal mutation or mutations, where “coupling linkage phase” refers to the fact that the favorable SNP allele was on the same homologous chromosome with the true favorable causal variant. The SNP in *KPNA3* that was most significant for BMW and LMW (Table 2) had lower frequency of the favorable allele in the fast-growing group than in the slow-growing one. This result would exclude this SNP from being a causal SNP but could be explained by the assumption that this SNP was in repulsion linkage phase with the true favorable causal variant.

**Table 3:** Frequencies of favorable alleles of 15 SNP markers in the 1.5 Mb GGA1 region of 173.5–175 Mb.

SNP	Position	Allele	FA	WRR	WRR1	XH	BEH
GGaluGA054833	173504098	C/T	C	0.83(80)	0.77(78)	0.27(79)	0.24(58)
rs15497877	173593810	C/T	C	0.52(80)	0.80(79)	0.38(79)	0.24(58)
**rs15497910**	173613981	A/G	A	0.38(80)	0.76(79)	0.44(79)	0.78(59)
GGaluGA054930	173841982	C/T	C	0.95(80)	0.96(79)	0.59(77)	0.53(58)
rs13972304	173931557	A/C	A	1.00(80)	1.00(79)	0.38(79)	0.44(59)
GGaluGA054970	173993933	T/G	G	0.66(80)	0.55(79)	0.17(79)	0.14(59)
rs13553164	174027867	C/T	T	0.64(80)	0.51(77)	0.07(77)	0.02(59)
rs14917305	174093115	C/T	C	0.79(70)	0.64(77)	0.06(79)	0.18(59)
**GGaluGA055001**	174122198	A/G	G	0.59(80)	0.27(79)	0.74(79)	0.51(57)
rs14917647	174379124	C/A	A	0.66(80)	0.41(79)	0.05(79)	0.06(59)
rs13553485	174594379	C/A	C	1.00(80)	0.94(79)	0.71(79)	0.44(58)
**GGaluGA055291**	174783129	C/T	C	0.61(80)	0.51(79)	0.54(79)	0.76(59)
rs13973515	174847719	C/T	T	0.57(80)	0.69(78)	0.25(79)	0.05(59)
GGaluGA055359	174921993	A/G	A	0.87(80)	0.99(79)	0.35(79)	0.38(59)
GGaluGA055379	174961349	C/T	C	0.66(80)	0.56(79)	0.26(79)	0.36(59)

FA = favorable allele for fast growing. Number in parentheses was the sample size. Bold face markers had unexpected frequencies for the favorable allele, lower in WRR and WRR1 and higher in XH and BEH.

The allele frequencies in fast-growing and slow-growing breeds showed that no SNP had a unique allele in any one breed. The SNP with the most striking allele frequency difference was at position 173,931,557 bp at GGA1 that most significant for ADG42. The favorable allele of this SNP was fixed in WRR and WRR1 and had frequencies of 0.38–0.44 (Table 3). Given that no breed-specific SNP was detected, the differences in growth traits likely involved more than one QTL. Results of LD analysis also favored to the possibility of multiple QTL for the growth traits. We should caution that the frequency estimates were based on targeted SNP genotyping without information about the chromosome-wide allele frequency data so that the possibility that the study population and the selected breeds for the targeted frequency estimates would have chromosome-wide frequency differences. The main utility of the targeted frequency analysis is that the frequency results were consistent with the hypothesis that the regions were associated with body weight.

**Figure 2:**
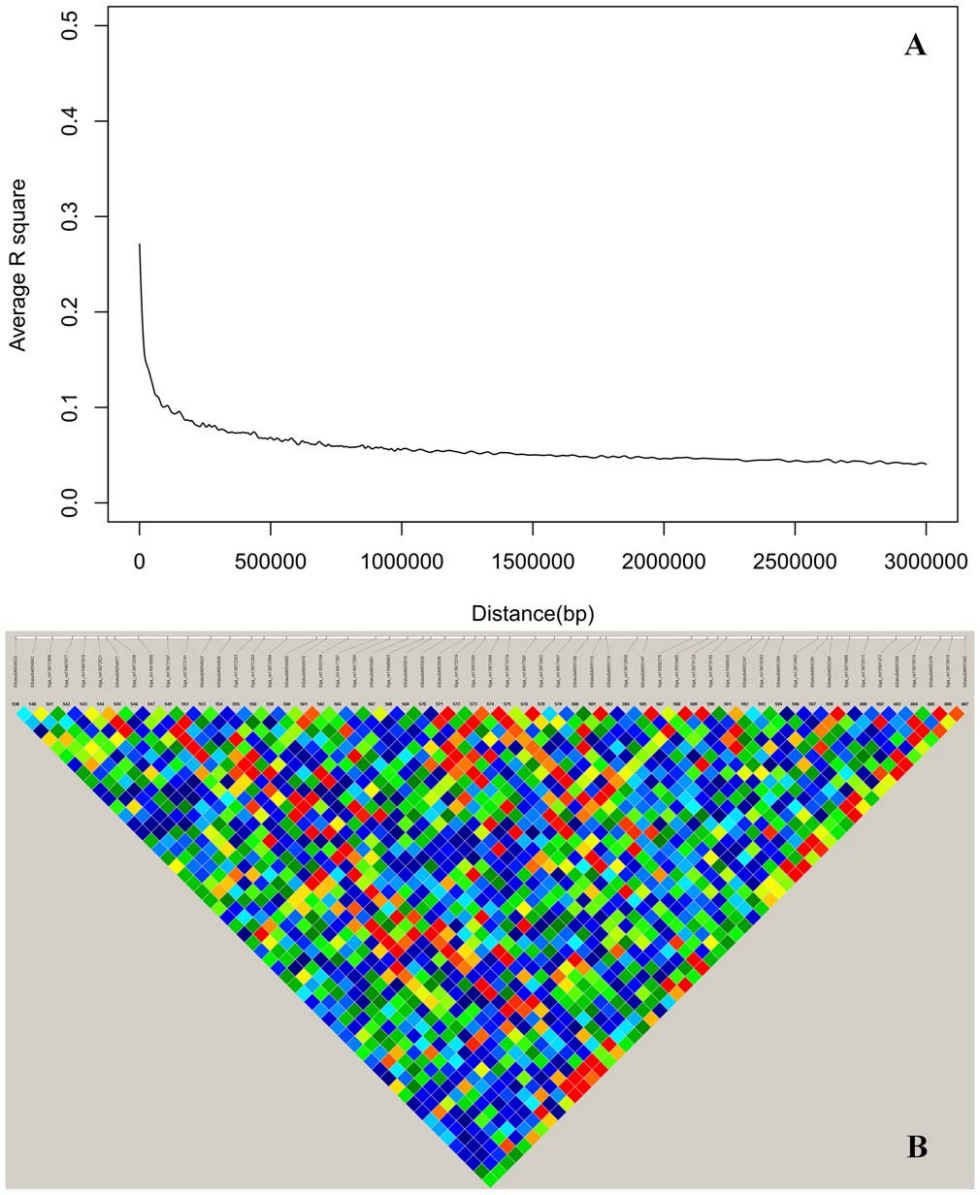
Linkage disequilibrium of GGA1. A. Linkage disequilibrium pattern of the F2 population from White Recessive Rock and Xinghua chickens crossing on GGA1. B. Linkage disequilibrium between SNPs in the 1.5 Mb GGA1 region of 173.5–175 Mb. Strongest LD signals were in red and weakest LD in blue.

Analysis of LD showed that LD intensity of GGA1 was weak in the F2 population crossed by WRR and XH. Strong LD in GGA1 was between loci approximately in 100 Kb distances, and LD declined to the background level at about 500 Kb distances (Figure 2A). In the 167–179 Mb region of GGA1 with all 257 significant SNP effects, LD signals were also weak, including LD values in the 1.5 Mb region of 173.5–175 Mb (Figure 2B). These results indicated that the significant SNP effects were likely due to multiple QTL rather than linked effects of a single QTL.

## Discussion

### Potential candidate genes

The 3 Mb region of 172–175 Mb with 63 coding genes and two microRNA genes had the most significant effects for all 23 traits (Figure 1) and appeared to be the most promising regions for candidate genes associated with chicken growth. Within this region, two known genes and an area lacking gene information had or were close to the most significant effects for all 23 traits, *FOXO1A* for ADG28, *KPNA3* for BMW and LMW, and the 20.9 Kb region downstream of *ENSGALG00000022732* for ADG42 (Table 2).

The *FOXO1A* gene, also called *FOXO1* in human or *Foxo1* in mouse, is a member of the FOXO forkhead type transcription factors. Although the specific function of *FOXO1A* has not yet been determined, this gene may play a role in myogenic growth and differentiation [21]–[26]. Overexpressing *Foxo1* transgenic mice would weigh less than the wild type mice and had a reduced skeletal muscle mass, and the muscle was paler in color due to red muscle reduction [21]. Similar results were observed in rats [22]. The *FOXO1A* gene had two SNPs in intron regions but none of these two markers was highly significant for any growth trait. The two highly significant SNPs were at 8.9 Kb upstream and 1.9 Kb downstream of *FOXO1A*, raising question whether a regulatory mechanism was involved in the significant SNP effects near *FOXO1A*. In human, the highest mRNA expression of *KPNA3* was in skeletal muscle in Genenote data [27].

The most significant SNP for ADG42 was in a region lacking gene information. This region had one NCBI gene (*LOC770248*), and two Ensembl genes (*ENSGALG00000017013* and *ENSGALG00000022732*). No biological information was available for these three genes. Allele frequency estimates showed that the SNP was fixed for the favorable allele in fast-growing WRR and WRR1 and had relative low frequencies of 0.38–0.44 in slow-growing XH and BEH. These frequency results favored the hypothesis that a causal mutation for chicken growth existed near the SNP, although none of the three genes nearest to this SNP had known biological functions. The next closest gene to the SNP was the *ribonuclease H2 subunit B* gene (*RNASEH2B*; 86.7 Kb upstream), which was known to specifically downgrade RNA [28], noting that two microRNA genes were about 413 Kb upstream of this gene.

**Figure 3:**
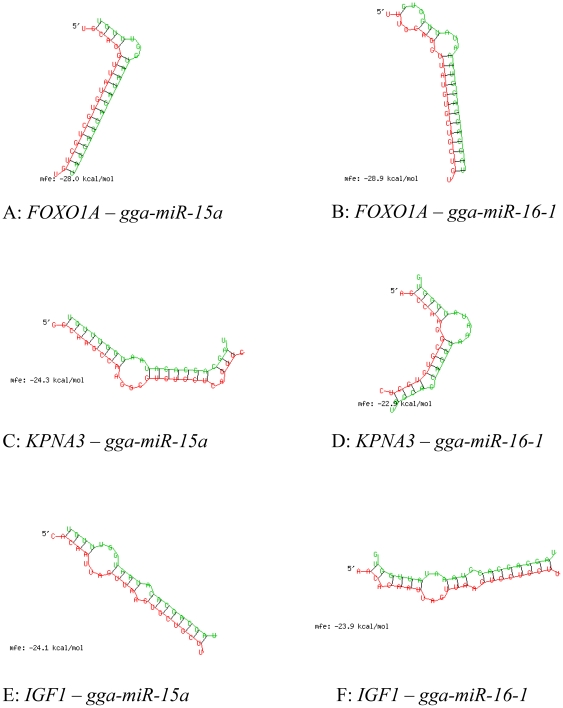
Molecular interactions between microRNAs and three prime untranslated regions (3′UTR) of *FOXO1A*, *KPNA3* and *IGF1*. Red letters indicate the 3′ UTR sequences of the target genes. Green letters indicate the matured sequences of *gga-miR-15a* or *gga-miR-16-1*.

The two microRNA genes, *gga-miR-15a* and *gga-miR-16-1*, approximately were in 300 Kb∼1 Mb distances to the five most significant SNPs in the 1.5 Mb region. MicroRNA genes are post-transcriptional regulators that result in translational repression and gene silencing by binding to complementary sequences on target messenger ribonucleic acids (mRNAs) in animals and human [29]. These two microRNAs, *gga-miR-15a* and *gga-miR-16-1*, were known to target some key genes such as *B-cell leukemia/lymphoma 2* to regulate tumor growth [30], [31]. *Insulin-like growth factor 1* (*IGF1*) gene, which involved in mediating growth and development, had a conserved binding site with *miR-15* and *miR-16* family in human [32], [33]. We investigated whether *IGF1*'s conserved binding with the two microRNA genes also existed in chickens and whether the two microRNA genes could have a gene regulation role by targeting certain coding genes in the 3 Mb region with 63 coding genes by bioinformatics prediction of molecular interactions requiring minimal free energy (MFE) *<*−20 calculated by RNAhybrid [34]. The results showed that chicken *IGF1* had a conserved binding site with *gga-miR-15a* and *gga-miR-16-1* (MFE = −24.1 and −23.9, Figure 3). This is interesting because *IGF1* is well-known for its roles in mediating growth and development. Both *gga-miR-15a* and *gga-miR-16-1* could bind to the mRNAs of *FOXO1A* and *KPNA3* (MFE = −28 and −28.9 for *FOXO1A* and MFE = −24.3 and −22.9 for *KPNA3*, Figure 3). None of the other 61 coding genes in the 3 Mb region could be confirmed as target genes by all three prediction tools. These results indicated some specificity of RNA targeting to *FOXO1A* and *KPNA3* by *gga-miR-15a* and *gga-miR-16-1*.

Combining results of RNA analysis and association analysis, the 3 Mb region of 172–175 Mb with 63 coding genes and two microRNA genes likely contained more than one causal mutations affecting chicken growth and could contain a gene regulatory mechanism. The 1.5 Mb region of *KPNA3*-*FOXO1A* could be immediate interest for candidate genes that may include *FOXO1A*, *KPNA3*, *INTS6*, *gga-miR-15a*, *gga-miR-16-1* and *RNASEH2B*. The only concern in declaring these loci as candidate genes was the fact that the favorable allele of *KPNA3* had lower frequency in WRR than in XH, although WRR1 had higher frequency than XH (0.76 vs. 0.44, Table 3). Two Ensembl coding genes (*ENSGALG0000002273* and *ENSGALG00000017013*) and a NCBI gene (*LOC770337*) between *gga-miR-16-1* and *RNASEH2B* could not be excluded as potential functional units affecting chicken growth because they were close to highly significant SNP effects, although these three genes had unknown biological functions.

### Comparison with previous results

Results in this study identified novel candidate genes in a 3 Mb GGA1 region and provided strong confirmation of some previously reported QTL effects. A number of studies reported significant QTL effects in the 167–179 Mb region of GGA1. Uemoto et al. also detected QTL affecting BW42 and BW63 in 165–172 Mb on GGA1 [11], and QTL effects were also reported in these regions were associated with BW28–BW84, average daily gain, WW or BMW [2], [6], [7], [10], [11], [15]. Besnier et al. reported a QTL for BW56 in the 169–175 Mb region of GGA1 with the most significant QTL effect at position 173,709,609 bp on GGA1 [35]. In the vicinity of this QTL peak we detected several highly significant SNP effects. The most significant effect for ADG42 was at 173,910,687 bp (Figure 1 and Table S1), 201 Kb downstream of the QTL peak in Besnier et al. [34]. Results from our study along with results in other studies represented strong evidence that the 167–179 Mb region, particularly the 172–175 Mb region, of GGA1 were strongly associated with growth in chickens. However, the results in this study differed from studies that reported QTL on other chromosomes. A recent study using 229 F2 chickens based on the crossing between the Silky breed and WPR reported SNP effects for growth traits in 7–12 wks in the 71.6–80.2 Mb GGA4 region and did not identify any significant SNP in the GGA1 region reported in this study [36]. In contrast, this study identified some SNP effects in the 71.6–80.2 Mb GGA4 region with lowered significance levels (*P* = 3.20×10^−07^∼3.62×10^−05^, Table S1), so that this study had some degree of confirmation of the GGA4 results. Breed difference (XH×WRR in this study and Silky×WPR in [36]) could be the main reason for the lack of confirmation by the study in [36] for the GGA1 results in this study. Although these two studies did not confirm each other for the GGA1 region, each had confirmation from results in the literature. Therefore, results in these two studies should add evidence to the process of achieving consensus for chicken growth QTL.

## Materials and Methods

### Ethics Statement

All of the animal experiments were conducted in accordance with regulations for the Administration of Laboratory Animals of Guandong Province. Animal experiments were approved by the Animal Care Committee of South China Agricultural University (Guangzhou, People's Republic of China) with approval number SCAU#0005.

### Experimental animals

An F2 design resource population was employed in the present study. The F2 resource population was derived from reciprocal crosses between WRR and XH chickens. WRR chicken is a fast-growing broiler breed and XH chicken is a slow-growing Chinese indigenous breed. Nine females and seven males from XH, eight females and nine males form WRR were selected for mating on the basis of consistent egg laying and semen production. Each male was paired with a female from the other line, except one male from XH, which paired with two females from WRR. Reciprocal mating of the XH (*♂*)×WRR (*♀*) and WRR (*♂*)×XH (*♀*) were selected on the basis of satisfactory egg and semen yields to produce the F1 generation. At 30 wk of age, 17 F1 males and 17 F1 females were selected to produce the F2 generation, resulting in a total of 489 birds in 17 full-sib families from six hatches at two-week intervals. The F2 individuals were raised in floor pens and fed commercial corn-soybean diets that met NRC requirements. Body weights were measured in grams at hatching, 7, 14, 21, 28, 35, 42, 49, 56, 63, 70, 77, 84, and 90 d of age (BW0, BW7, BW14, BW21, BW28, BW35, BW42, BW49, BW56, BW63, BW70, BW77, BW84, and BW90). The ADG was calculated based on the difference between the current BW and the BW of two weeks ago for 1–14, 15–28, 29–42, 43–56, 57–70, and 71–84 d of age (ADG14, ADG28, ADG42, ADG56, ADG70, and ADG84). For example, ADG28 was calculated as (BW28 – BW14)/14 and represented the net daily weight increase during the period of 15–28 d. All 489 F2 individuals (252 males and 237 females) were slaughtered and measured for BMW (g), LMW (g), and WW (g) at 90 d of age [13], [37].

### SNP selection and genotyping

Genomic DNA was extracted from vein blood samples using saturated phenol-chloroform extraction method. Thirty-three F0, 32 F1, and 489 F2 individuals were quantified for DNA concentrations and genotyped using the 60 K SNP Illumina iSelect chicken array developed by USDA Chicken GWMAS Consortium, Cobb Vantress, and Hendrix Genetics, containing more than 57,000 SNPs [38], [39] with average spacing 17.9 Kb. This 60 K SNP chip is a multi-sample genotyping panel supported by Illumina's Infinium® II Assay. SNPs were distributed on GGA1–28, GGA32, and chromosome Z, W, mitochondria, and two linkage groups: LGE22C19-W28_E50C23 (from here on called LGE22) and LGE64. To evaluate genotyping reliability, 26 DNA samples randomly selected out of 554 samples were genotyped twice, and over 99.96% concordance rate of called genotypes was obtained. SNP selection required less than 5% missing genotypes, less than 2% non-Mendelian error rate, 95% or more call rate, 1% minor allele frequency, and Hardy-Weinberg equilibrium (*P>*0.00001). As a result of these SNP selection criteria, 47,678 SNPs were selected for use in the GWAS. Distribution of the 47,678 SNPs by chromosome is presented in Table S3. Genotyping of the SNPs was carried out by DNA LandMarks Incorporation (Quebec, Canada).

### Statistical and bioinformatics analyses

Statistical tests of SNP-phenotype association were implemented using the generalized least square version of epiSNP computer package that considered sib correlation within each family [40], [41]. The statistic model was,
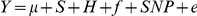
where *Y* = corrected phenotypic value, *μ* = common mean, *S* = fixed gender effect, *H* = fixed hatch effect, *f* = random family effect, *SNP* = the single-locus SNP genotypic effect, and *e* = random residual. Additive and dominance effects were tested using linear contrasts of the single-locus SNP genotypic effect [41]. Body weights except BW0, BW14, BW21, BW63 and BW90 had slight deviations from normality and Box-Cox and Johnoson transformations implemented by Minitab 15 [42] were used to achieve normality. ADG traits had normal distributions and untransformed ADG values were used in the association tests.

Genome-wide significance was defined based on the “LD adjusted” Bonferroni method [43] to correct p-value thresholds at three levels of significance: suggestive association (1 time of false positive per GWAS), significant association (0.05 false positives per GWAS) that we used as “genome-wide significance”, and highly significant association (0.001 false positives per GWAS). The F2 population was estimated to have 24,522 “independent” tests (Table S4) based on the “solid spine of LD” algorithm with a minimum D′ value of 0.8 calculated by Haploview [44]. Therefore, the three significant threshold P-values were 4.08×10^−5^ (1/24,522) for suggestive significance, 2.04×10^−6^ (0.05/24,522) for genome-wide significance and 4.08×10^−8^ (0.001/24,522) for “highly significant”. Overview of SNP effects by Manhattan plots were produced by SNPEVG version 2.1 [45].

Bioinformatics prediction of molecular interactions between microRNAs and three prime untranslated regions of the coding genes used RNA22 [46], RNAhybrid [34] and TargetScan [47], [48], requiring binding from all three methods. Minimal free energy (MFE)*<*−20 calculated by RNAhybrid was required for reporting binding. Gene locations were based on Ensembl [49] and NCBI [28].

### Allelic frequency spectrum analyses

Four random chicken populations, WRR, WRR1, XH, and BEH, were used for analyzing allelic frequency spectrum among breeds. WRR1 was a fast-growing chicken line from a commercial company in Guangdong, China. Both XH and BEH were slow-growing and from Gongdong, Jiangxi Province, China, respectively. Sample sizes of WRR, WRR1, XH and BEH were 80, 79, 79, and 59 birds, respectively. Sequenom technique platform was used for genotyping the 16 significant SNPs located in the 173.5–175 Mb region of GGA1. The SNP at 173,776,019 bp was fixed in WRR, WRR1 and BEH and had a high frequency of 0.92. We considered this SNP to have a high likelihood to be a monomorphic marker and removed this marker from frequency analysis, so that only 15 of the original 16 SNPs were used for frequency analysis. The four populations were subjected to primer extension and MALDI-TOF mass spectrometry using MassARRAY Compact System by Bioyong Technologies Incorporation (Beijing, China). SNPs were genotyped with the use of a commercially available Complete Genotyping Reagent Kit for MassARRAY® Compact 384 and ABI GeneAmp® 9700 384 Dual (Sequenom Inc., San Diego, California, USA), in accordance with the manufacturer's instructions. The sequence detection software, Typer 4.0, provided by Sequenom, was used for genotyping analysis.

## Supporting Information

Figure S1 **Manhattan plots for SNP effects for 23 growth traits.** Aggregate weekly body weight: BW0, BW7, BW14, BW21, BW28, BW35, BW42, BW49, BW56, BW63, BW70, BW77, BW84, BW90; Biweekly average daily gain: ADG14, ADG28, ADG42, ADG56, ADG70, ADG84; Brest muscle weigh: BMW; Leg muscle weight: LMW; and Wing weight: WW. The green solid line indicates genome-wide significance (*P<*2.04×10-6) with “LD adjusted” Bonferroni correction.
(PDF)

Table S1 **SNP effects with suggestive significance (** ***P<*** **4.08×10^−5^) for 23 growth traits.**
(XLSX)

Table S2 **Chi-square tests of frequency differences between fast- and slow-growing breeds for SNPs in the 173.5–175 Mb GGA1 region.**
(XLSX)

Table S3 **Distribution of SNPs by chromosome.**
(XLSX)

Table S4 **LD blocks in the F2 population.**
(XLSX)
