# Supplementary material for: Genome-Wide Association Study Identified a Narrow Chromosome 1 Region Associated with Chicken Growth Traits
Source: PLoS One. 2012 Feb 16;7(2):e30910. doi: 10.1371/journal.pone.0030910 (PMC3281030; doi:10.1371/journal.pone.0030910)

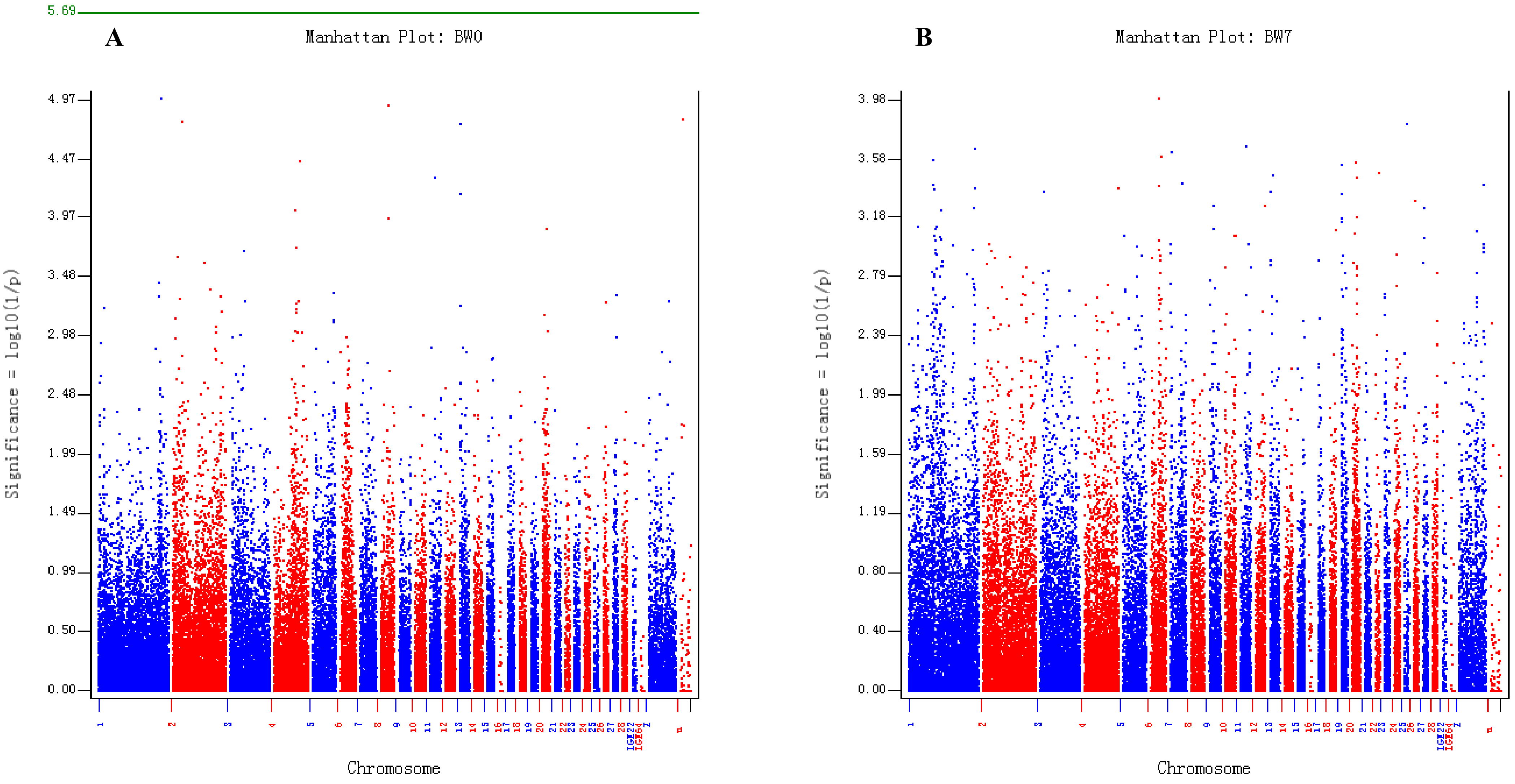
A
Manhattan Plot: BW0
5.69
4.97
4.47
3.97
3.48
2.98
2.48
1.99
1.49
0.99
0.50
0.00
Significance = log10(1/p)
Chromosome
B
Manhattan Plot: BW7
3.98
3.58
3.18
2.79
2.39
1.99
1.59
1.19
0.80
0.40
0.00
Significance = log10(1/p)
Chromosome

C

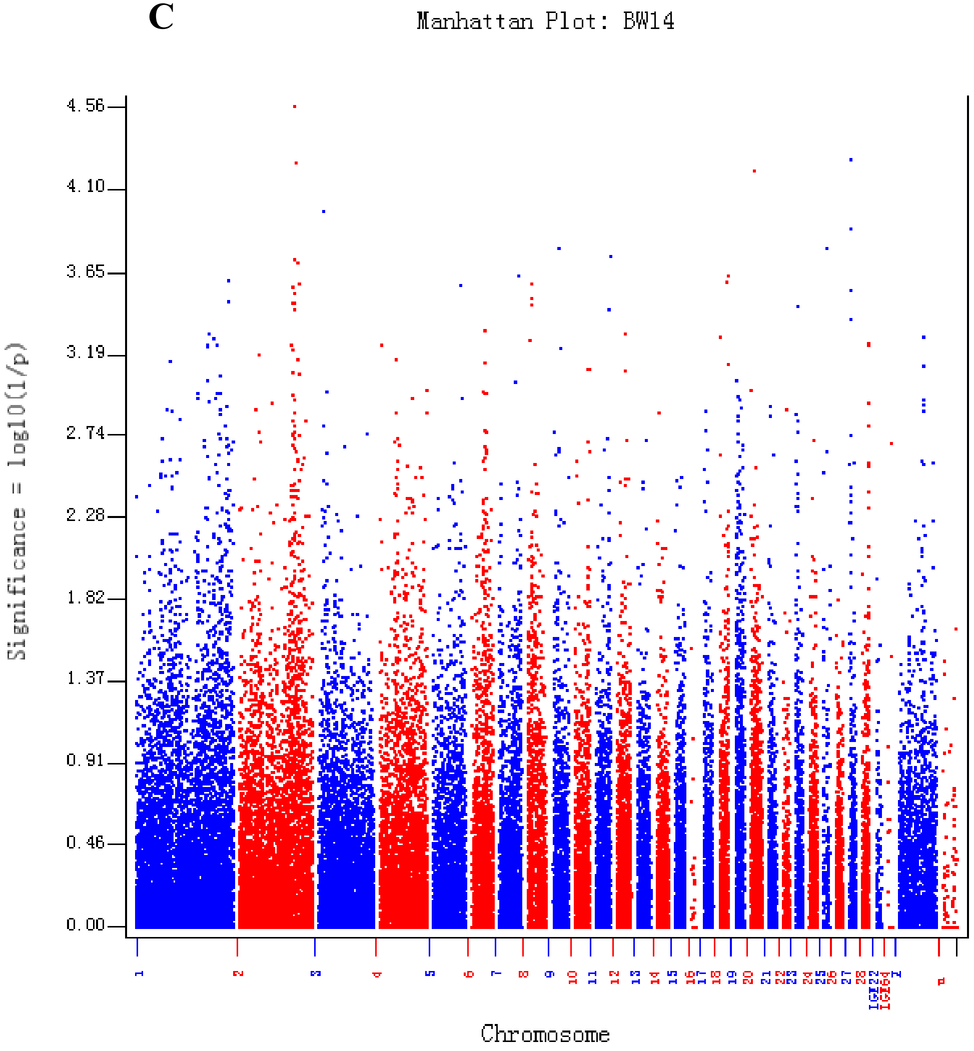
Manhattan Plot: BW14
Significance = log10(1/p)
4.56
4.10
3.65
3.19
2.74
2.28
1.82
1.37
0.91
0.46
0.00
Chromosome

D

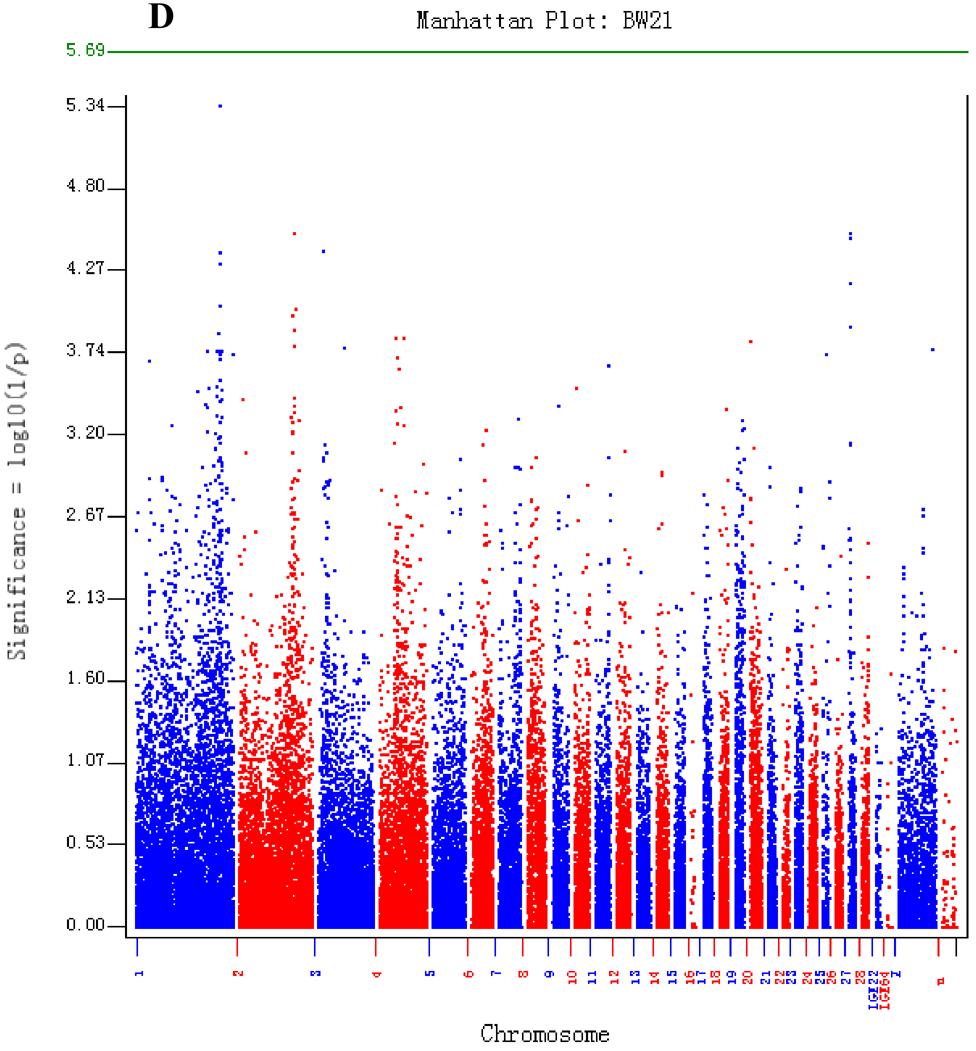
Manhattan Plot: BW21
5.69
Significance = log10(1/p)
5.34
4.80
4.27
3.74
3.20
2.67
2.13
1.60
1.07
0.53
0.00
Chromosome

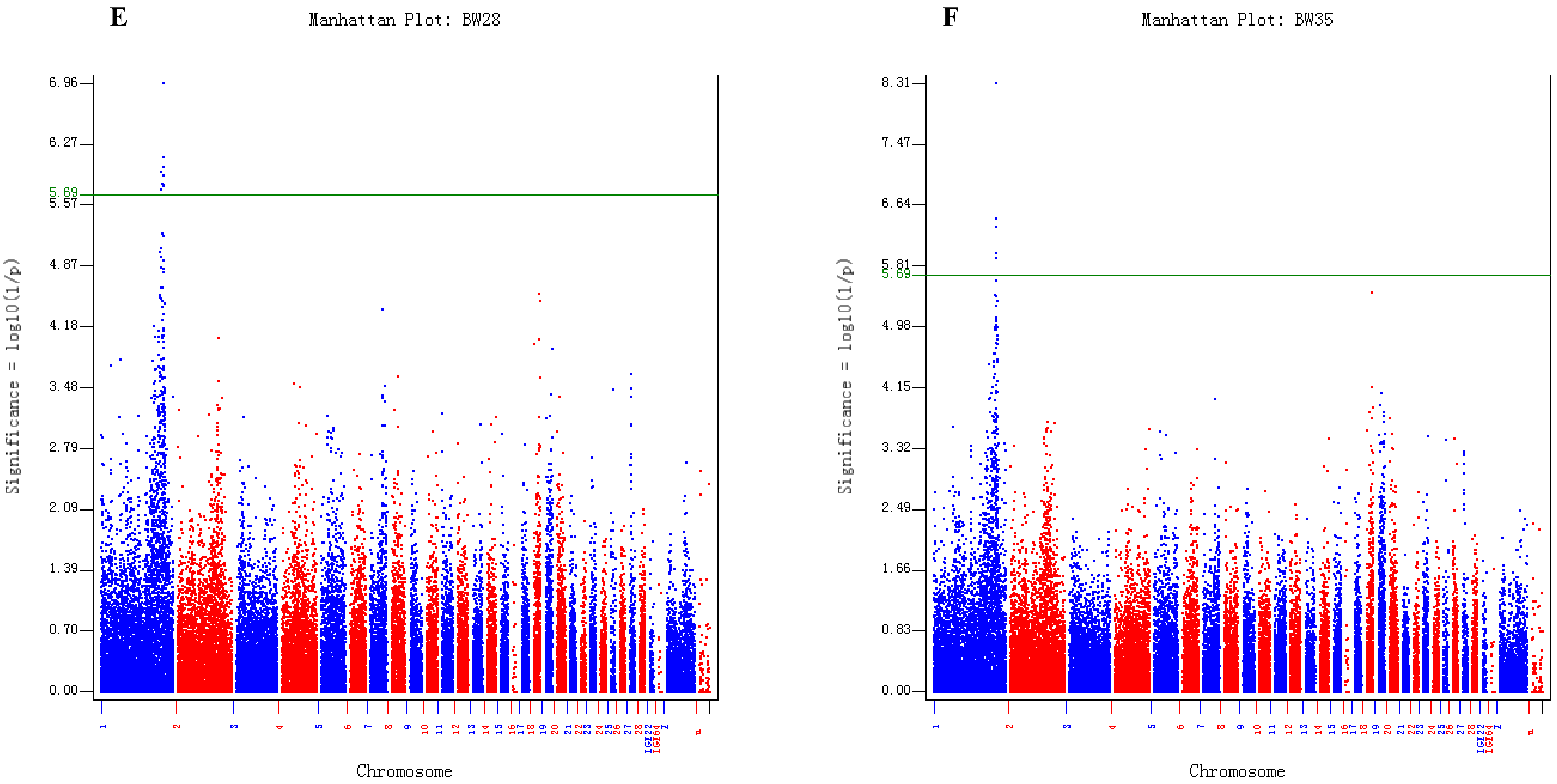
E
Manhattan Plot: BW28
6.96
6.27
5.69
5.57
4.87
4.18
3.48
2.79
2.09
1.39
0.70
0.00
Significance = log10(1/p)
Chromosome
F
Manhattan Plot: BW35
8.31
7.47
6.64
5.81
5.69
4.98
4.15
3.32
2.49
1.66
0.83
0.00
Significance = log10(1/p)
Chromosome

G

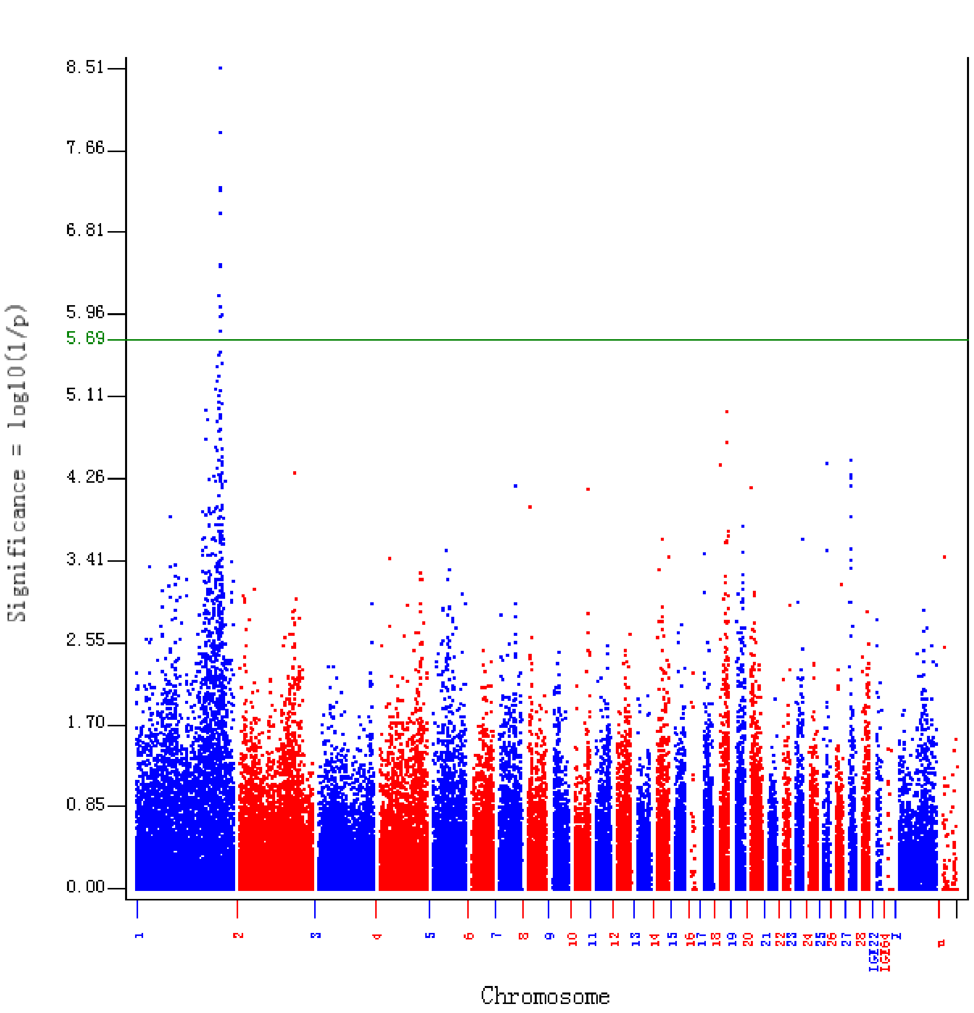
Manhattan Plot: BW42
Significance = log10(1/p)
8.51
7.66
6.81
5.96
5.69
5.11
4.26
3.41
2.55
1.70
0.85
0.00
Chromosome

H

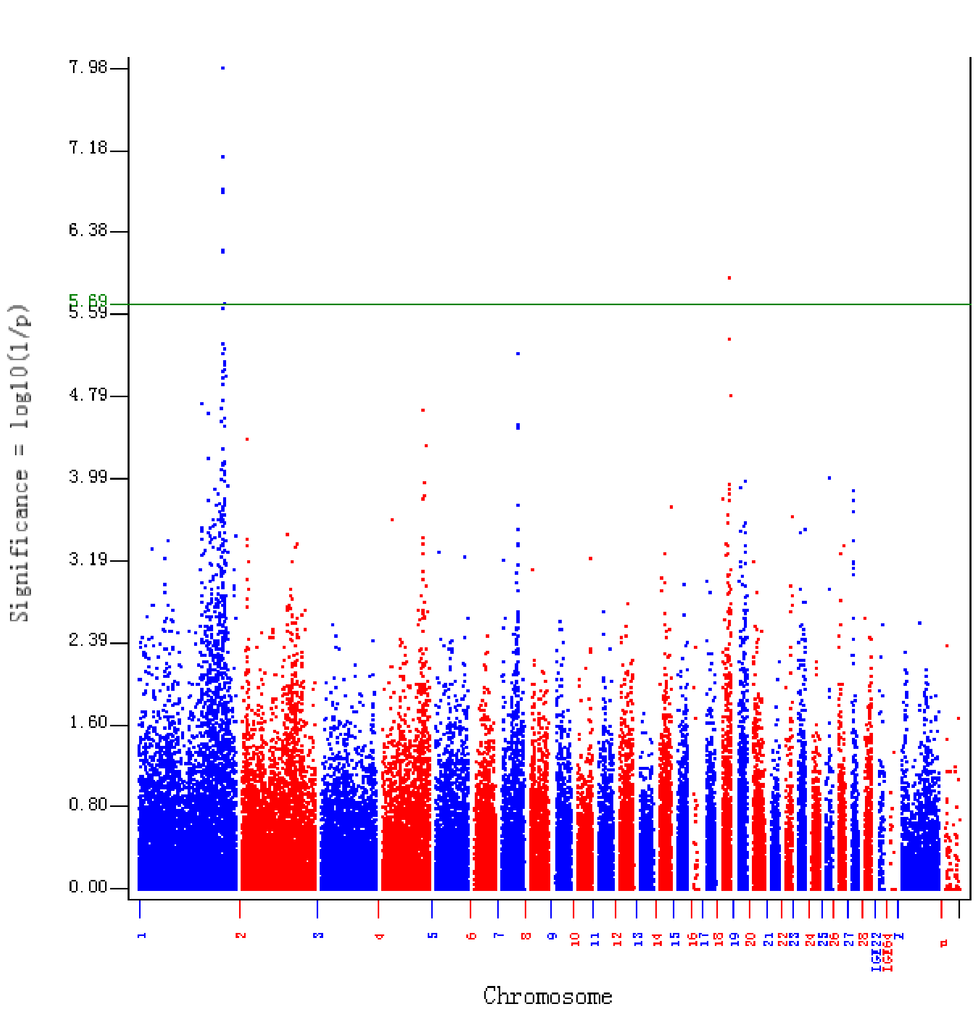
Manhattan Plot: BW49
Significance = log10(1/p)
7.98
7.18
6.38
5.69
5.59
4.79
3.99
3.19
2.39
1.60
0.80
0.00
Chromosome

I

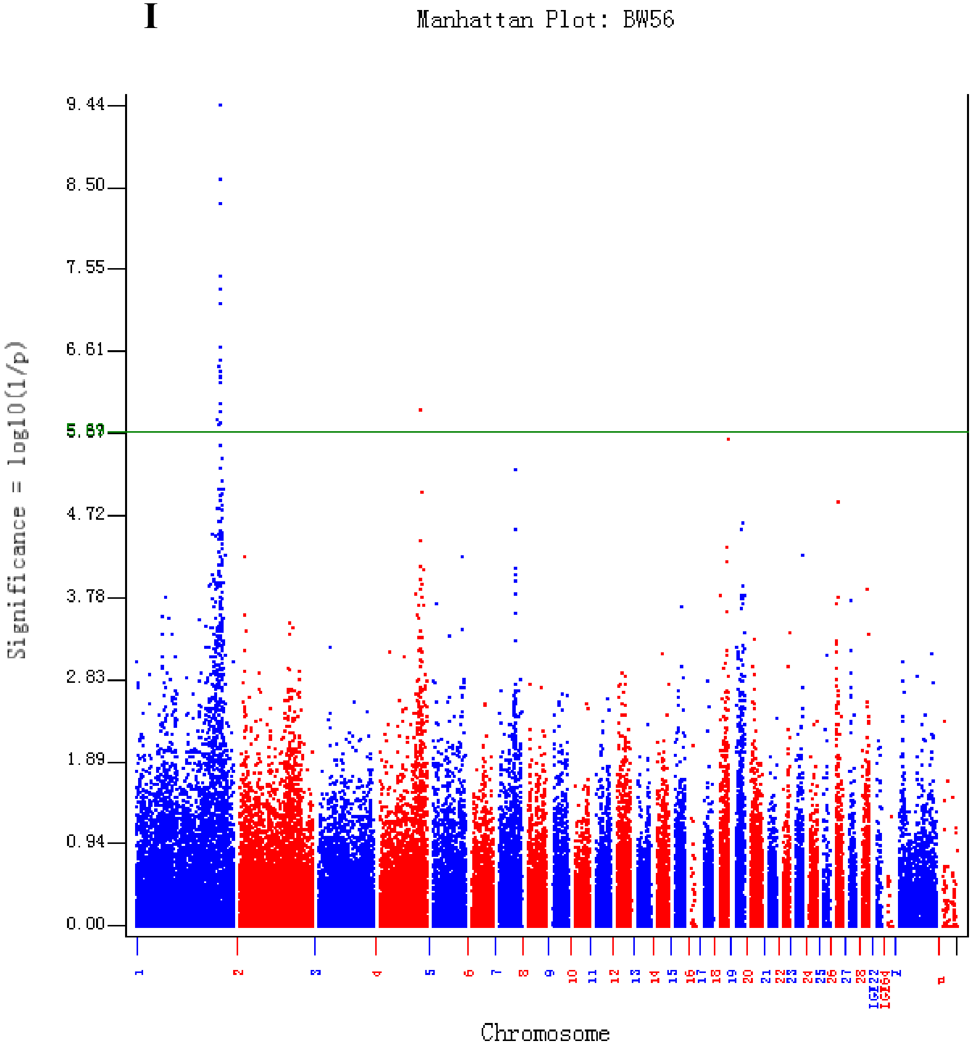
Manhattan Plot: BW56
9.44
8.50
7.55
6.61
4.72
3.78
2.83
1.89
0.94
0.00
Significance = log10(1/p)
Chromosome

J

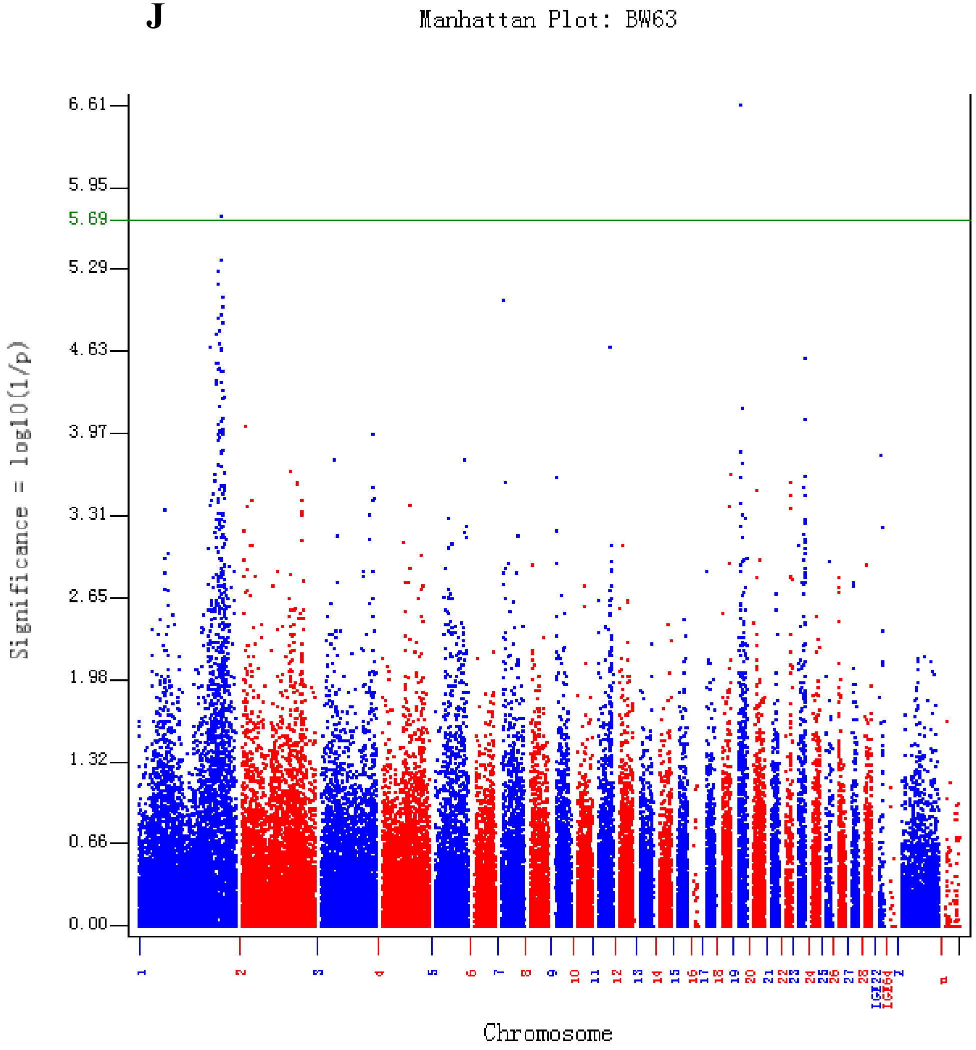
Manhattan Plot: BW63
6.61
5.95
5.69
5.29
4.63
3.97
3.31
2.65
1.98
1.32
0.66
0.00
Significance = log10(1/p)
Chromosome

K

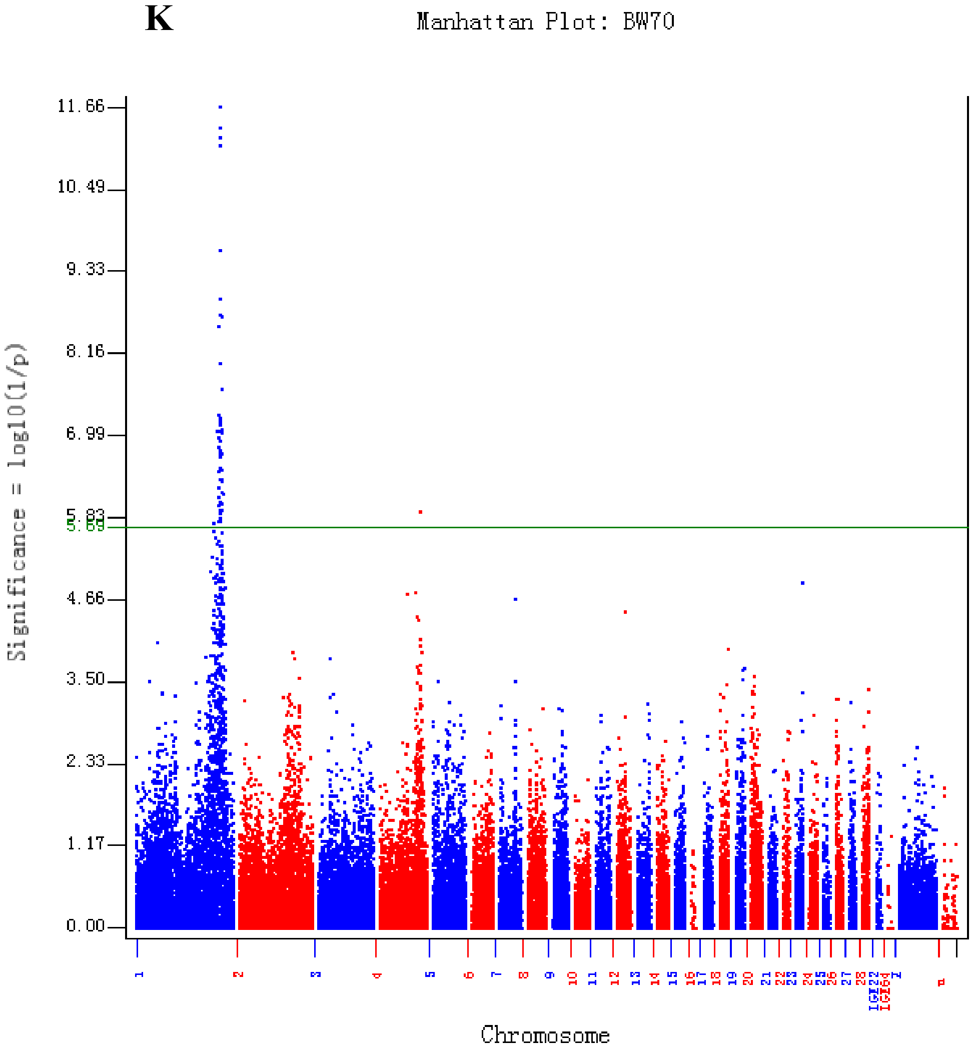
Manhattan Plot: BW70
Significance = log10(1/p)
11.66
10.49
9.33
8.16
6.99
5.83
5.69
4.66
3.50
2.33
1.17
0.00
Chromosome

L

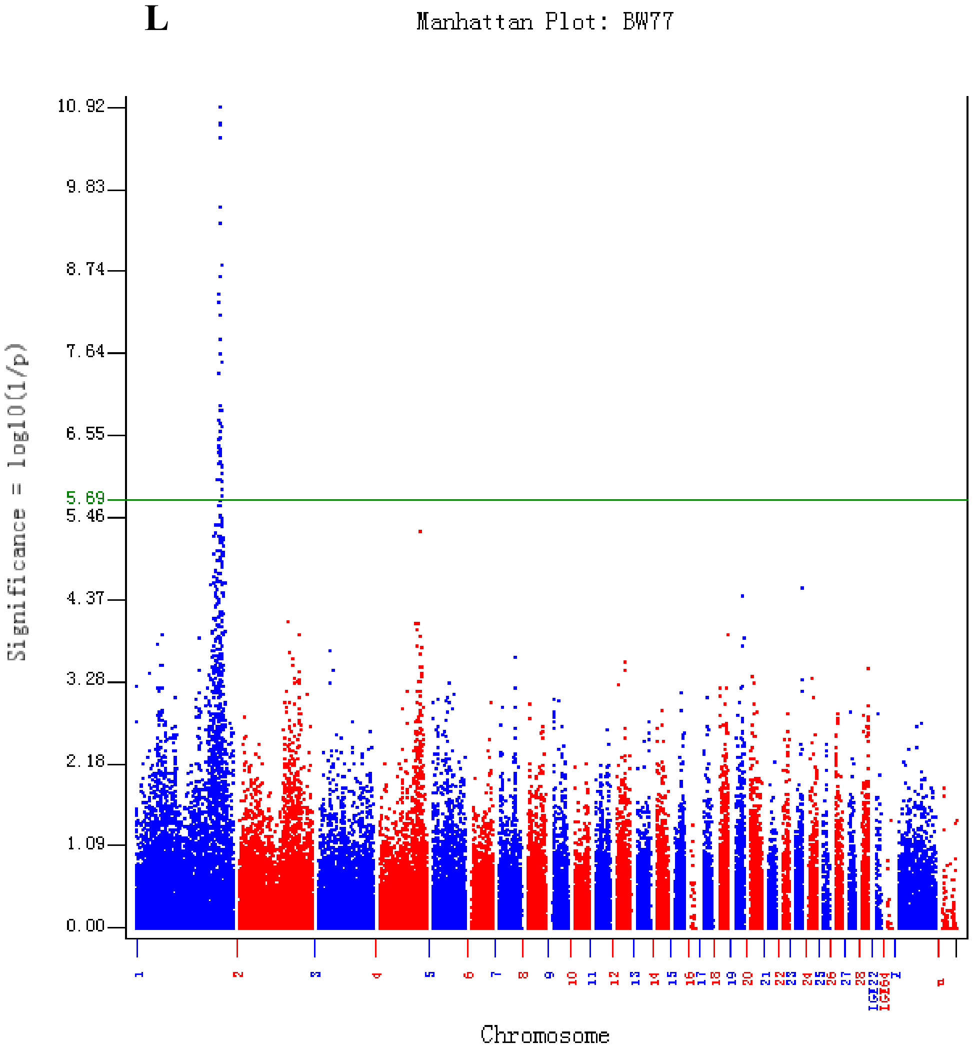
Manhattan Plot: BW77
Significance = log10(1/p)
10.92
9.83
8.74
7.64
6.55
5.69
5.46
4.37
3.28
2.18
1.09
0.00
Chromosome

M Manhattan Plot: BW84

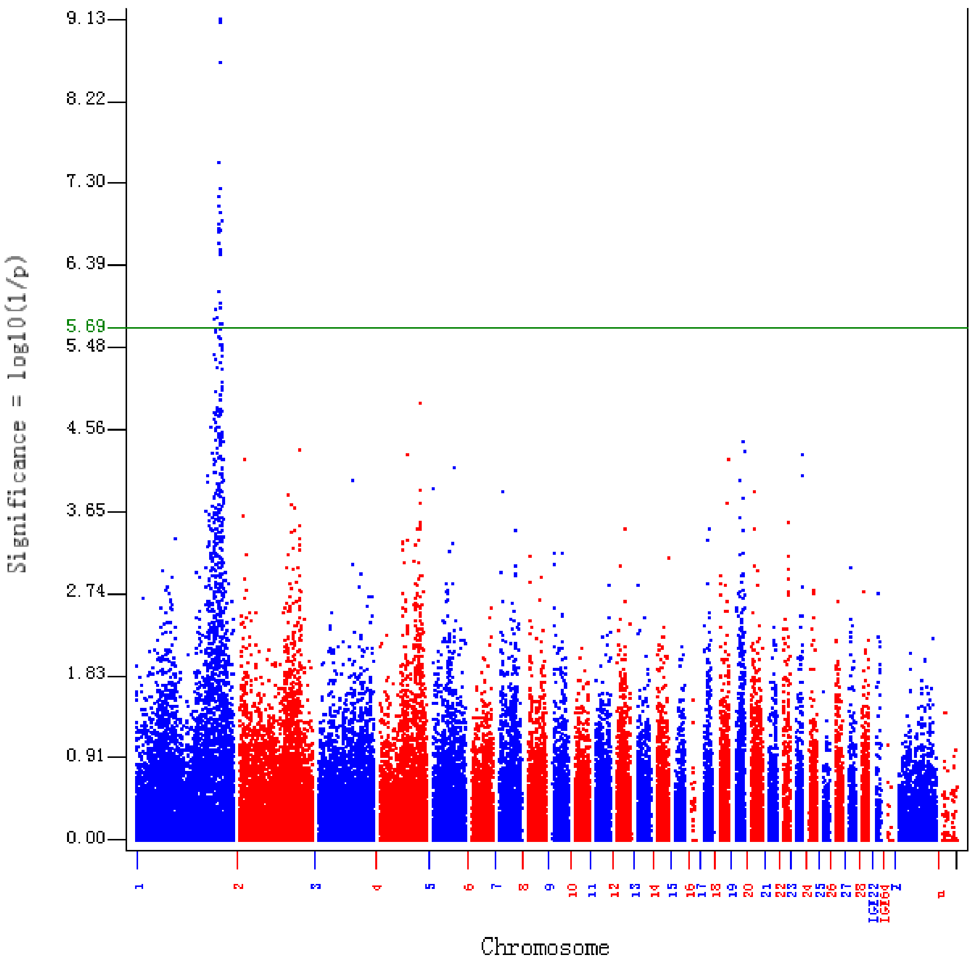

N Manhattan Plot: BW90

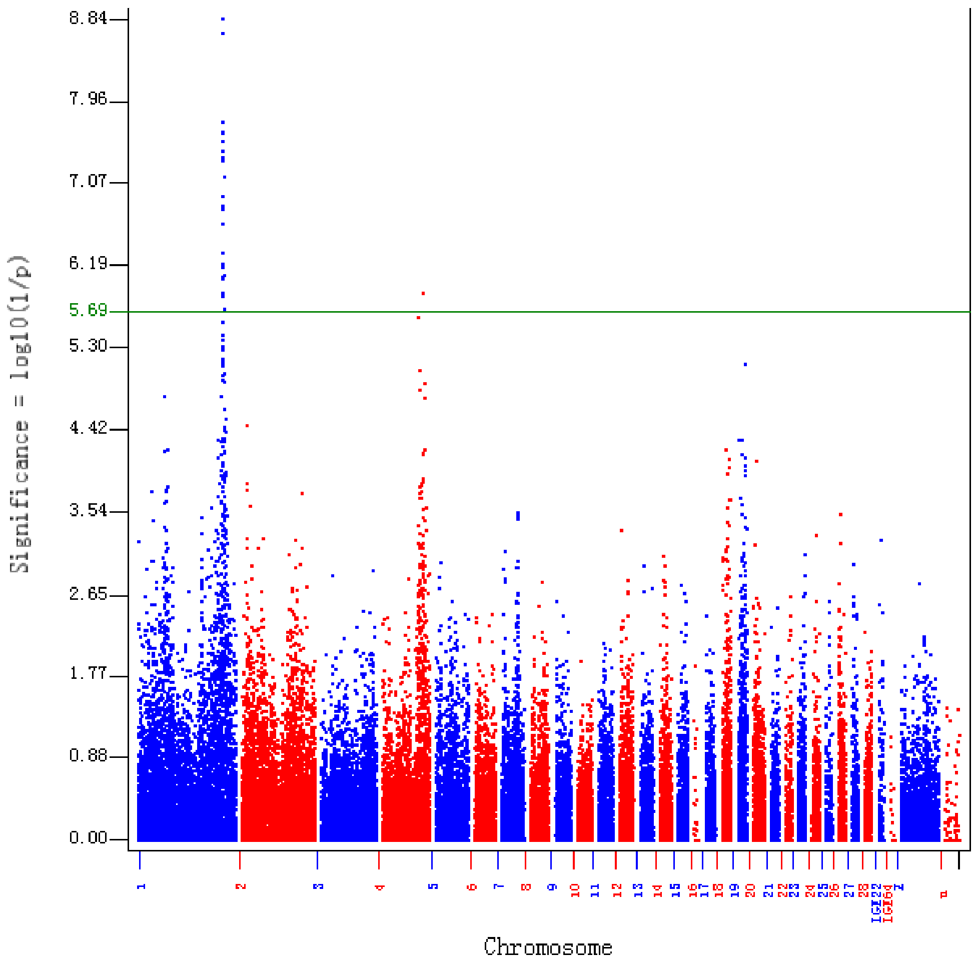

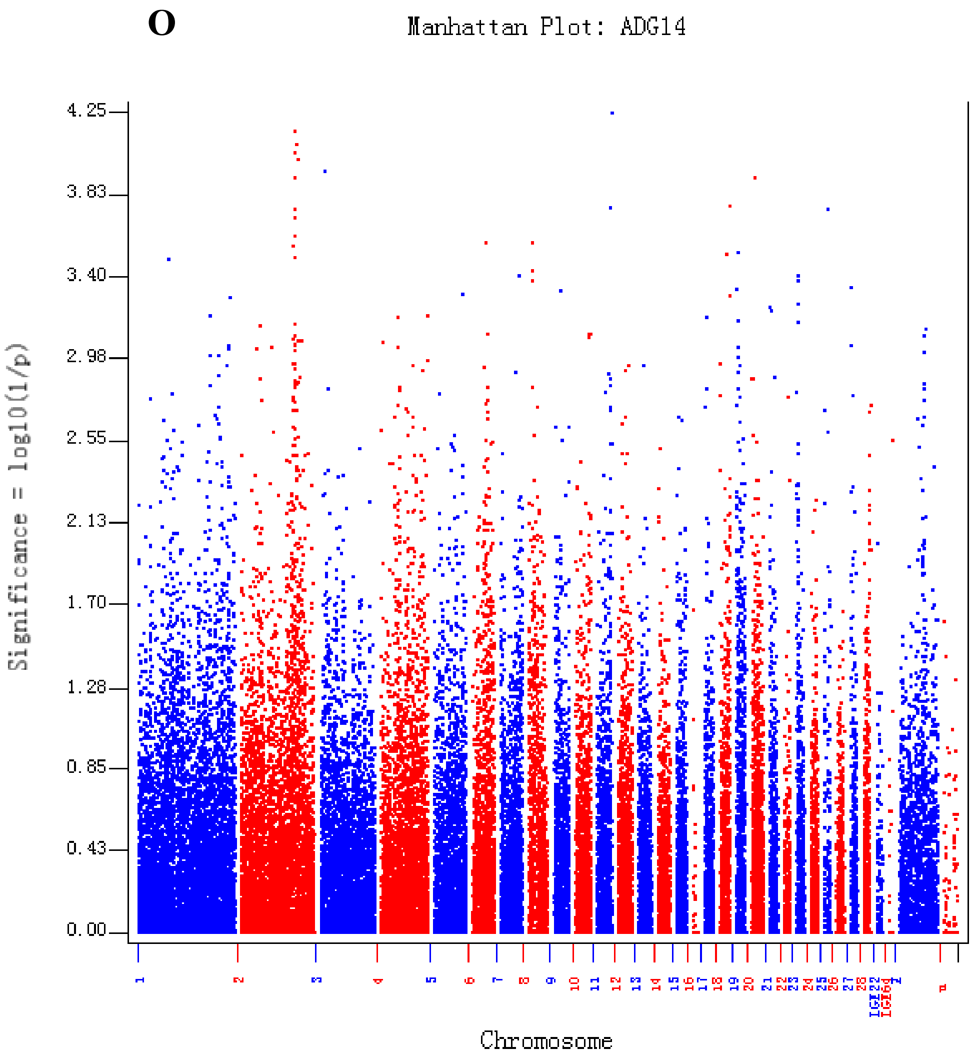
O
Manhattan Plot: ADG14
Significance = log10(1/p)
Chromosome

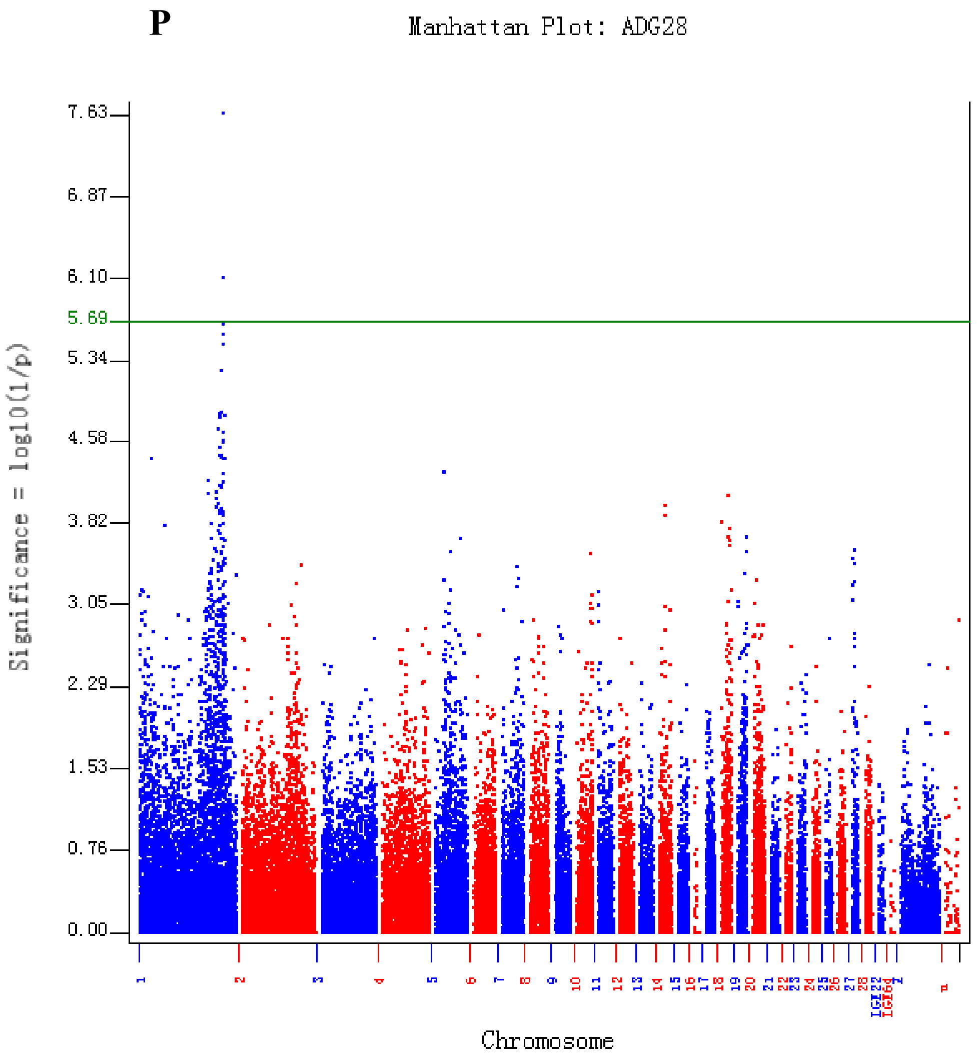
P
Manhattan Plot: ADG28
Significance = log10(1/p)
5.69
Chromosome

Q

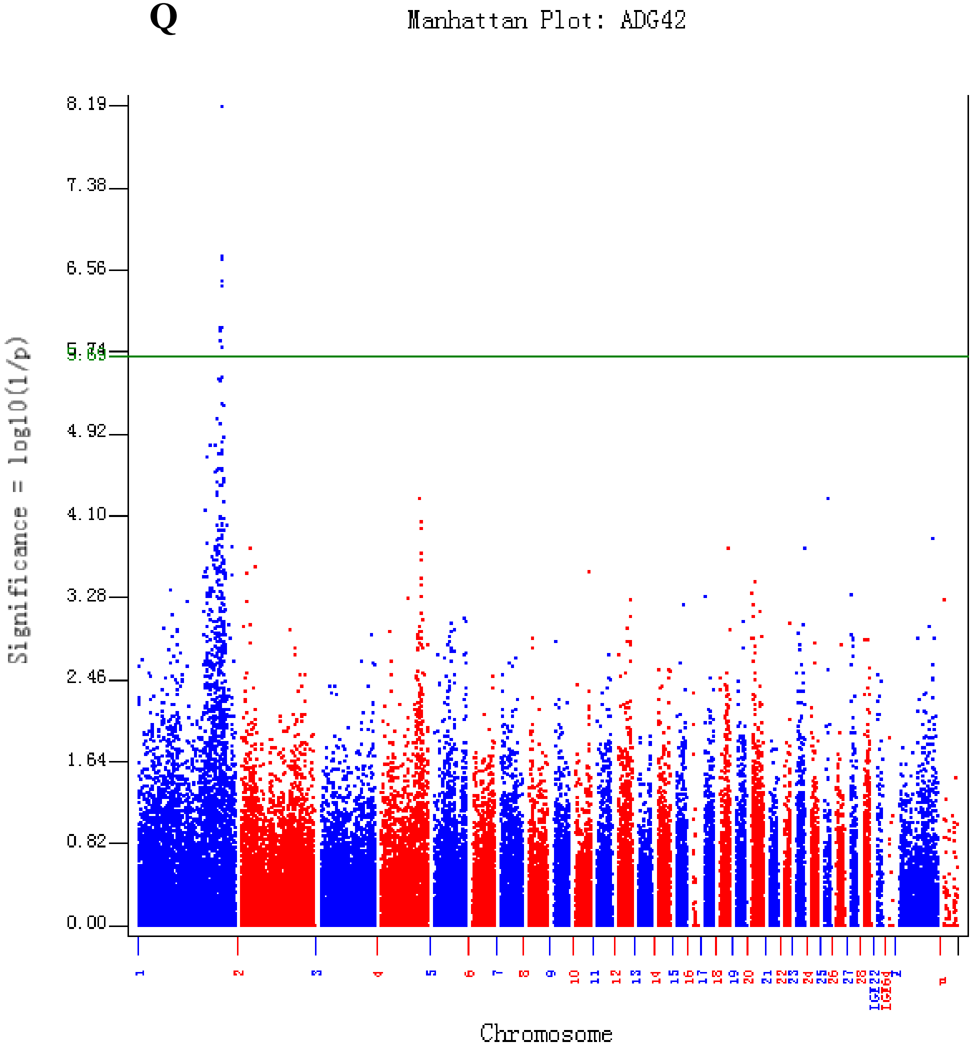
Manhattan Plot: ADG42
Significance = log10(1/p)
8.19
7.38
6.56
5.74
5.65
4.92
4.10
3.28
2.46
1.64
0.82
0.00
Chromosome

R

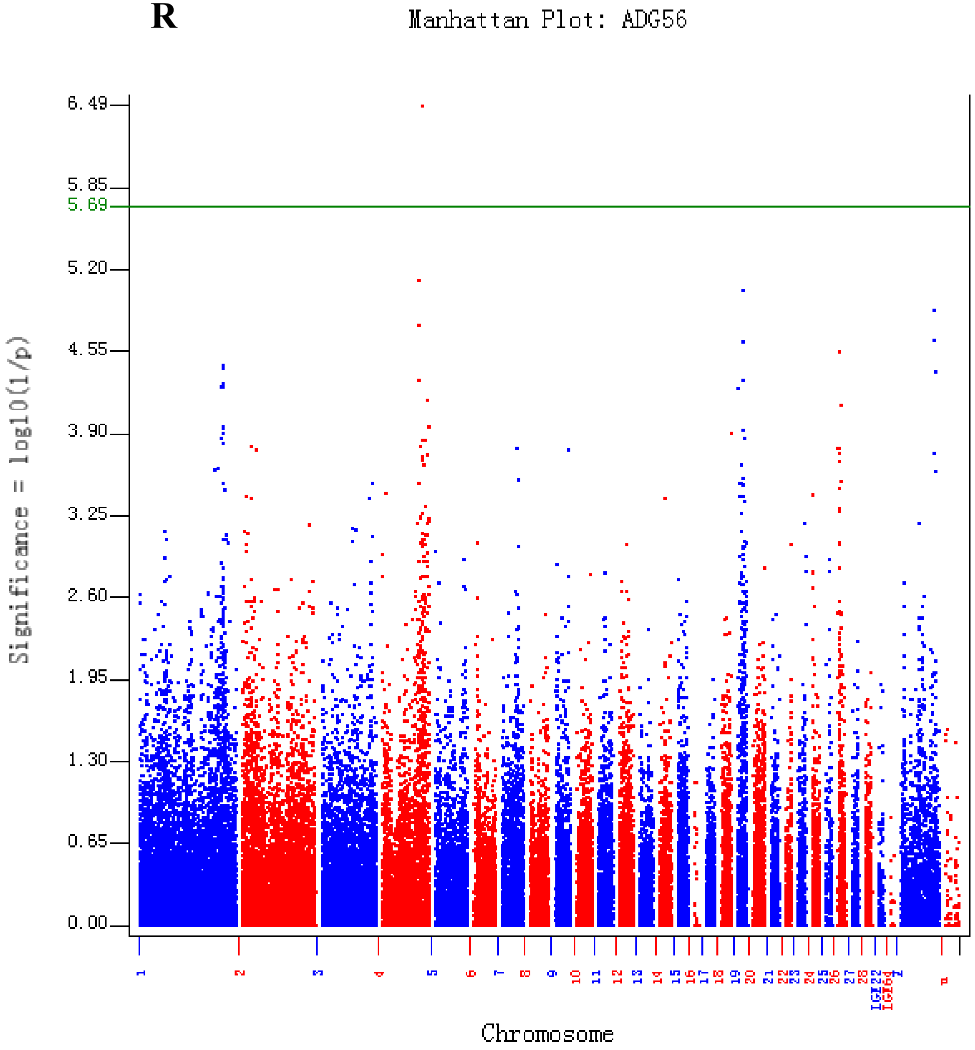
Manhattan Plot: ADG56
Significance = log10(1/p)
6.49
5.85
5.69
5.20
4.55
3.90
3.25
2.60
1.95
1.30
0.65
0.00
Chromosome

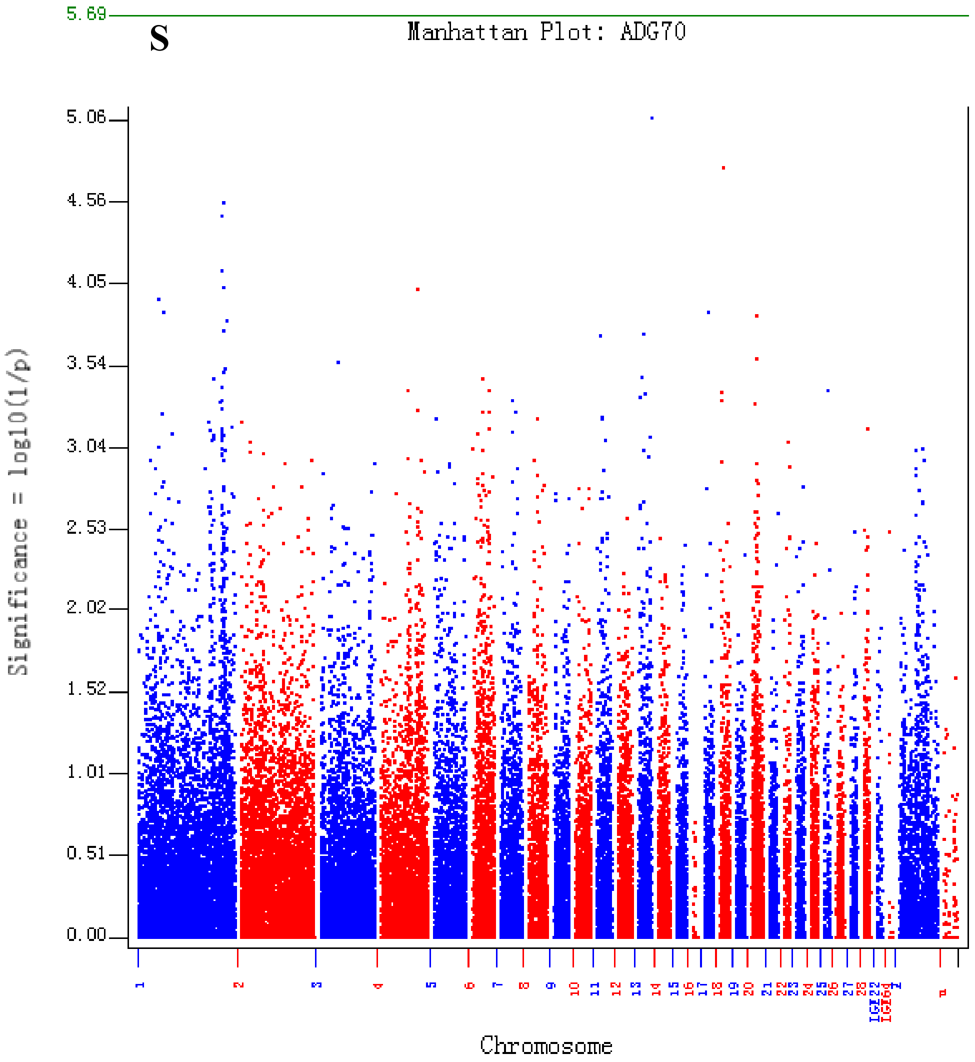
S
Manhattan Plot: ADG70
5.69
5.06
4.56
4.05
3.54
3.04
2.53
2.02
1.52
1.01
0.51
0.00
Significance = log10(1/p)
Chromosome

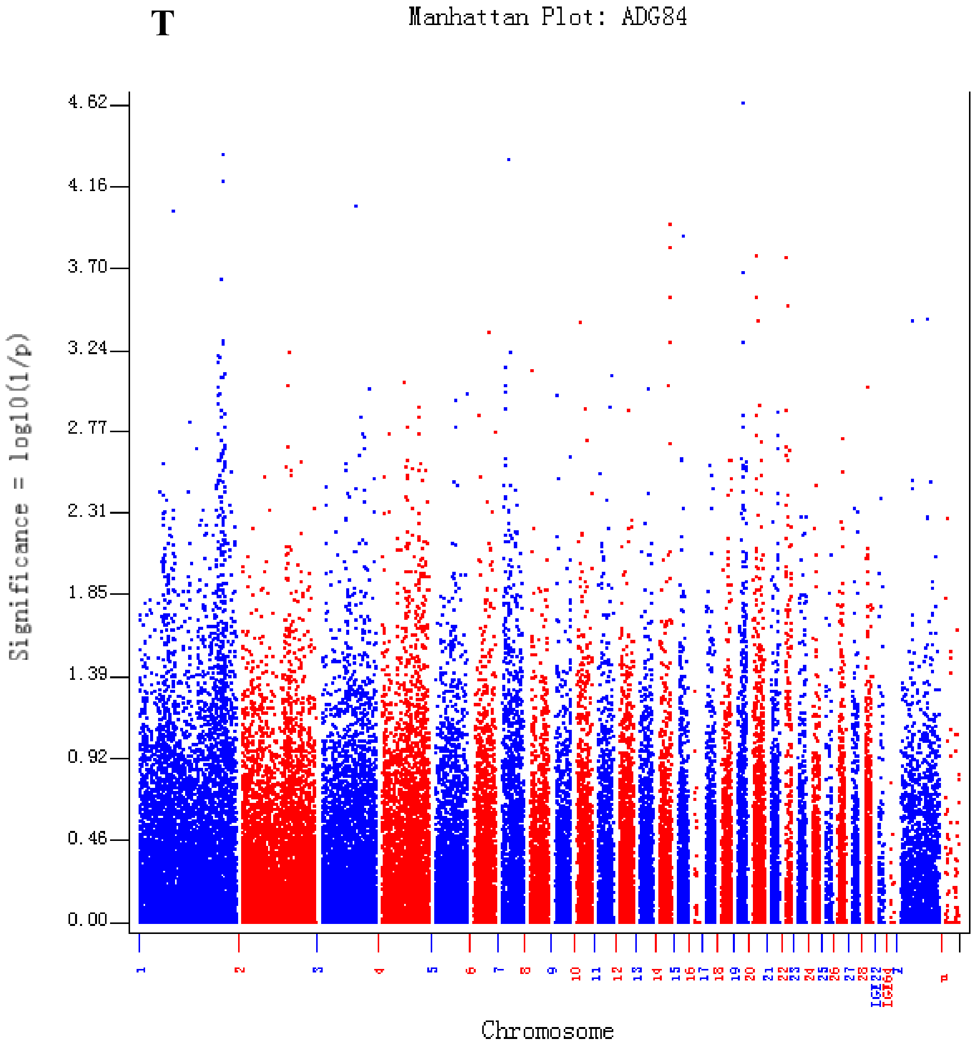
T
Manhattan Plot: ADG84
4.62
4.16
3.70
3.24
2.77
2.31
1.85
1.39
0.92
0.46
0.00
Significance = log10(1/p)
Chromosome

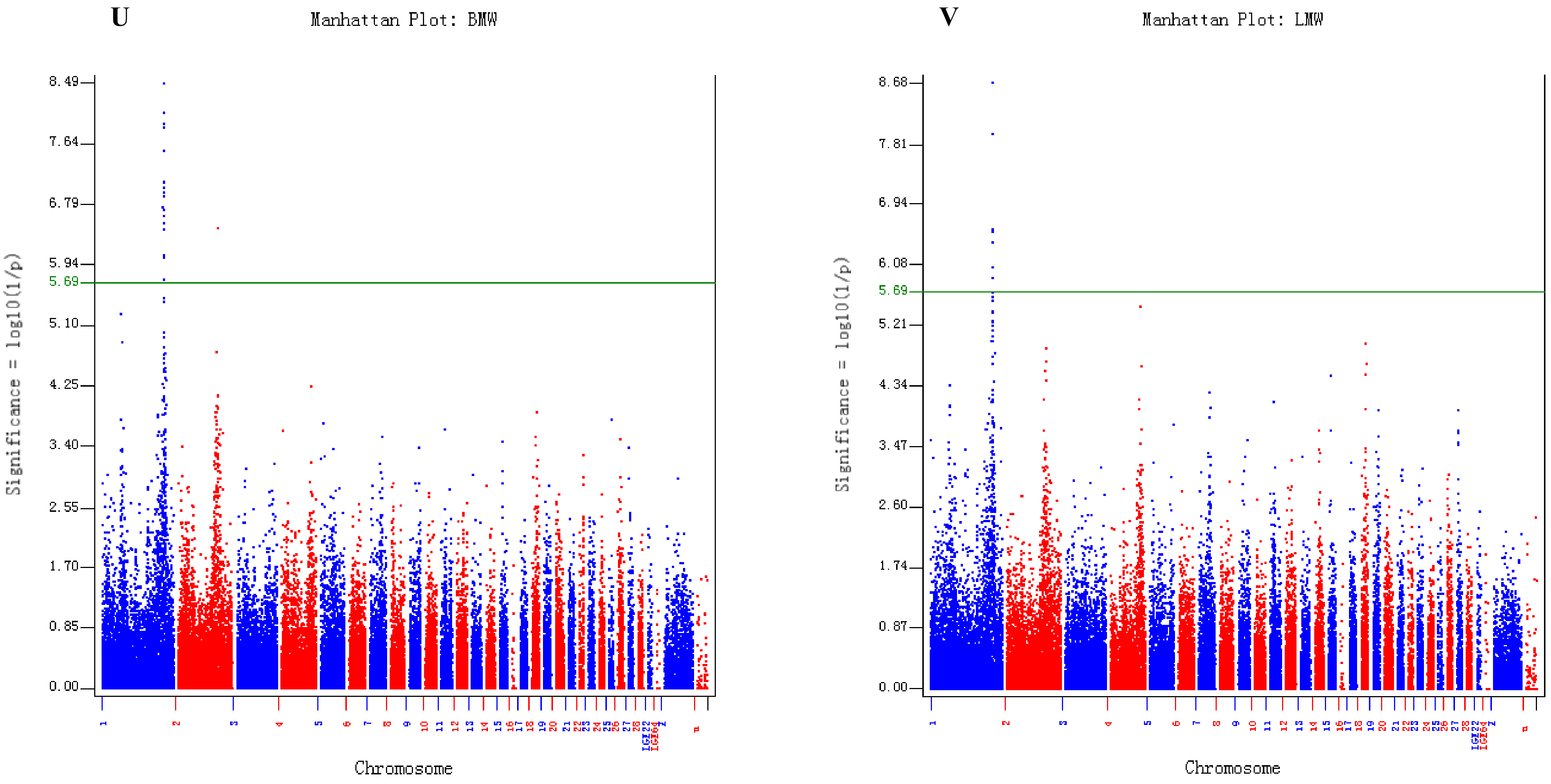
U
Manhattan Plot: BMW
Significance = log10(1/p)
8.49
7.64
6.79
5.94
5.69
5.10
4.25
3.40
2.55
1.70
0.85
0.00
Chromosome
V
Manhattan Plot: LMW
Significance = log10(1/p)
8.68
7.81
6.94
6.08
5.69
5.21
4.34
3.47
2.60
1.74
0.87
0.00
Chromosome

W

Manhattan Plot: WW

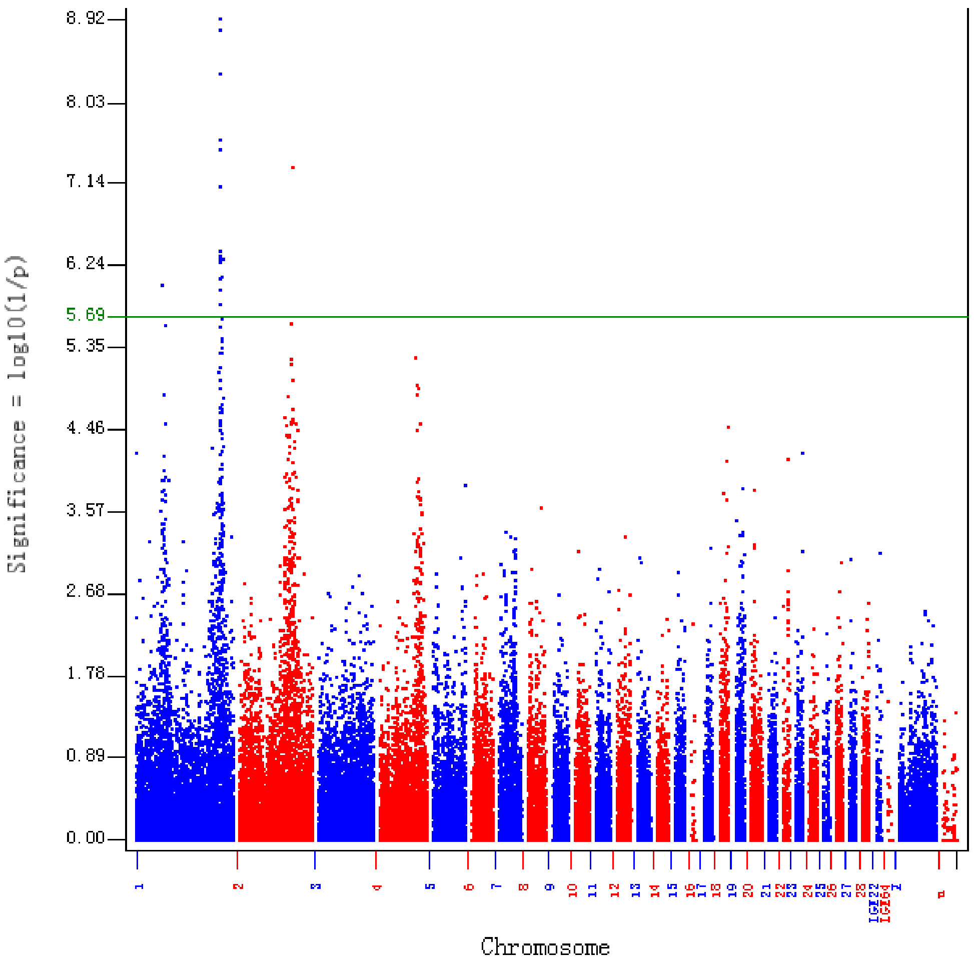

Supplement: Figure S1 — Manhattan plots for SNP effects for 23 growth traits. Aggregate weekly body weight: BW0, BW7, BW14, BW21, BW28, BW35, BW42, BW49, BW56, BW63, BW70, BW77, BW84, BW90; Biweekly average daily gain: ADG14, ADG28, ADG42, ADG56, ADG70, ADG84; Brest muscle weigh: BMW; Leg muscle weight: LMW; and Wing weight: WW. The green solid line indicates genome-wide significance (P<2.04×10^-6^) with “LD adjusted” Bonferroni correction. (PDF) [file pone.0030910.s001.pdf]
